# Ilizarov External Fixation: Percutaneous Gigli Saw Versus Multiple Drill-hole Osteotomy Techniques for Distraction Osteogenesis

**DOI:** 10.7759/cureus.4973

**Published:** 2019-06-22

**Authors:** Asadullah Makhdoom, Jagdesh Kumar, Adeel A Siddiqui

**Affiliations:** 1 Orthopaedic Surgery & Traumatology, Liaquat University of Medical & Health Sciences, Jamshoro, PAK; 2 Orthopaedic Surgery, Dow University of Health Sciences, Karachi, PAK

**Keywords:** distraction osteogenesis, gigli saw, multiple drill holes, osteotomy

## Abstract

Introduction

Many different methods and variations have been employed to perform osteotomy for deformity correction, bone lengthening, and segmental bone transport. Currently, multiple drill-hole (MDH) and Gigli saw osteotomies are the two most preferred ones, being favoured over other techniques. Our objective is to compare the modified healing index (mHI) of these two commonly used procedures.

Methodology

This retrospective study was conducted at the department of Orthopedics, Liaquat University of Medical and Health Sciences, Jamshoro, Pakistan. The study population consisted of all skeletally mature patients who underwent tibial bone osteotomy for bone lengthening or bone transport using Ilizarov circular fixator from June 2016 to September 2018. We excluded patients with metabolic bone disease and patients who underwent osteotomy for deformity correction. Preoperative and operative patients’ demographics and clinical data were gathered through a review of medical record and mHI was calculated to compare the effectiveness of osteotomy techniques.

Results

A total of 50 patients, 74% males and 26% females 26% with a mean age of 33.14 ± 12 years were included in the study. Of the 50 patients, 23 (27 osteotomies) had undergone MDH osteotomy (group I), whereas 27 patients (37 osteotomies) had a Gigli saw osteotomy (group II). The overall mHI of both groups was 1.60 ± 0.34 month/cm (range 1.0-2.5 month/cm). When we compared the mHI of both techniques, the mean mHI was 1.72 ± 0.33 month/cm (range 1.2 - 2.5 months/cm) in MDH group and 1.54 ± 0.36 month/cm (range 1.0-2.5 month/cm) in the Gigli saw group. The healing index was significantly lower in the Gigli saw group. None of our patients showed nonunion at the osteotomy site. However, the problems of incomplete osteotomy in two cases and bone fractures in four cases were seen in MDH osteotomy.

Conclusion

According to our results, percutaneous Gigli saw osteotomy technique by two small incisions minimizes the local soft tissue trauma and periosteal disruption around the osteotomy more than the multiple drill holes osteotomy, resulting in better consolidation following distraction osteogenesis.

## Introduction

Osteotomy was popularized by Professor Gavril Abramovich Ilizarov in the 1950s, and since that time, many different methods to perform osteotomy have been described for deformity correction, bone lengthening, and segmental bone transport [1-2]. An ideal osteotomy technique is one that minimizes soft tissue and periosteal disruption, lessens thermal necrosis, and produces high-quality healing tissue [1]. These goals can be accomplished with a technique that is truly minimally invasive and preserves the medullary, as well as periosteal blood supply [3-4].

Currently, multiple drill-hole (MDH) and Gigli saw osteotomy are the two foremost methods for osteotomy, being favoured and recommended by many orthopedic surgeons [3-6]. Recently, several studies have evaluated their outcome and reported the best results for quality of the regenerated bone in comparison with other methods of osteotomy [2,7].

Most of the published studies have evaluated the clinical and radiological outcome of Ilizarov fixator. There is a paucity of data comparing the difference in healing indices between percutaneous Gigli saw and MDH osteotomy techniques in patients undergoing distraction osteogenesis. Hence, the purpose of this study was to evaluate if there is a difference in the modified healing index (mHI) of percutaneous Gigli saw compared with MDH osteotomy.

## Materials and methods

This retrospective and quasi-experimental study with consecutive non-probability sampling of patients was undertaken at the department of Orthopedic Surgery, Liaquat University of Medical and Health Sciences, Jamshoro, Pakistan. The study population consisted of all skeletally mature patients who underwent tibial bone osteotomy for bone lengthening or transport using Ilizarov circular fixator, in a two year period from June 2016 to September 2018. We excluded patients with metabolic bone disease and those who underwent osteotomy for deformity correction. All osteotomies of tibial bone were performed by a senior orthopedic surgeon, by using the previously presented standard surgical technique.

Patients were divided into two groups; Group I included 23 patients in which MDH osteotomy was performed and Group II included 27 patients in which Gigli saw osteotomy was performed. In the post-operative period, all patients followed the same rehabilitation protocol and were reviewed bi-weekly during the distraction period, and then monthly until radiological signs of consolidation of the regenerated bone were observed.

Consolidation time was defined as the time from the beginning of distraction until the full strengthening of regenerated bone, defined by the formation of three of four complete cortices at least 2 mm in thickness of the regenerated bone in two orthogonal radiograms. Preoperative and operative patients’ demographics and clinical data including age, gender, surgical indications, technique of osteotomy, level of osteotomy, consolidation time, and follow-up duration were gathered through a review of the medical record. mHI (consolidation time per centimeter of lengthening instead of total time in the frame) was calculated to compare the effectiveness of osteotomy technique [7]. We used this modification because in some patients tibial bone osteotomy was already consolidated, but there was considerable delay in waiting for the transport docking site to unite.

The data were entered and analyzed in IBM Statistical Package for the Social Sciences Statistics for Windows, Version 22.0 (IBM Corp., Armonk, NY). Data are reported as mean and standard deviation (SD). Student t-test and Fisher's exact test was used to compare non-parametric means. A p-value of ≤0.05 was taken as significant.

## Results

**Figure 1:**
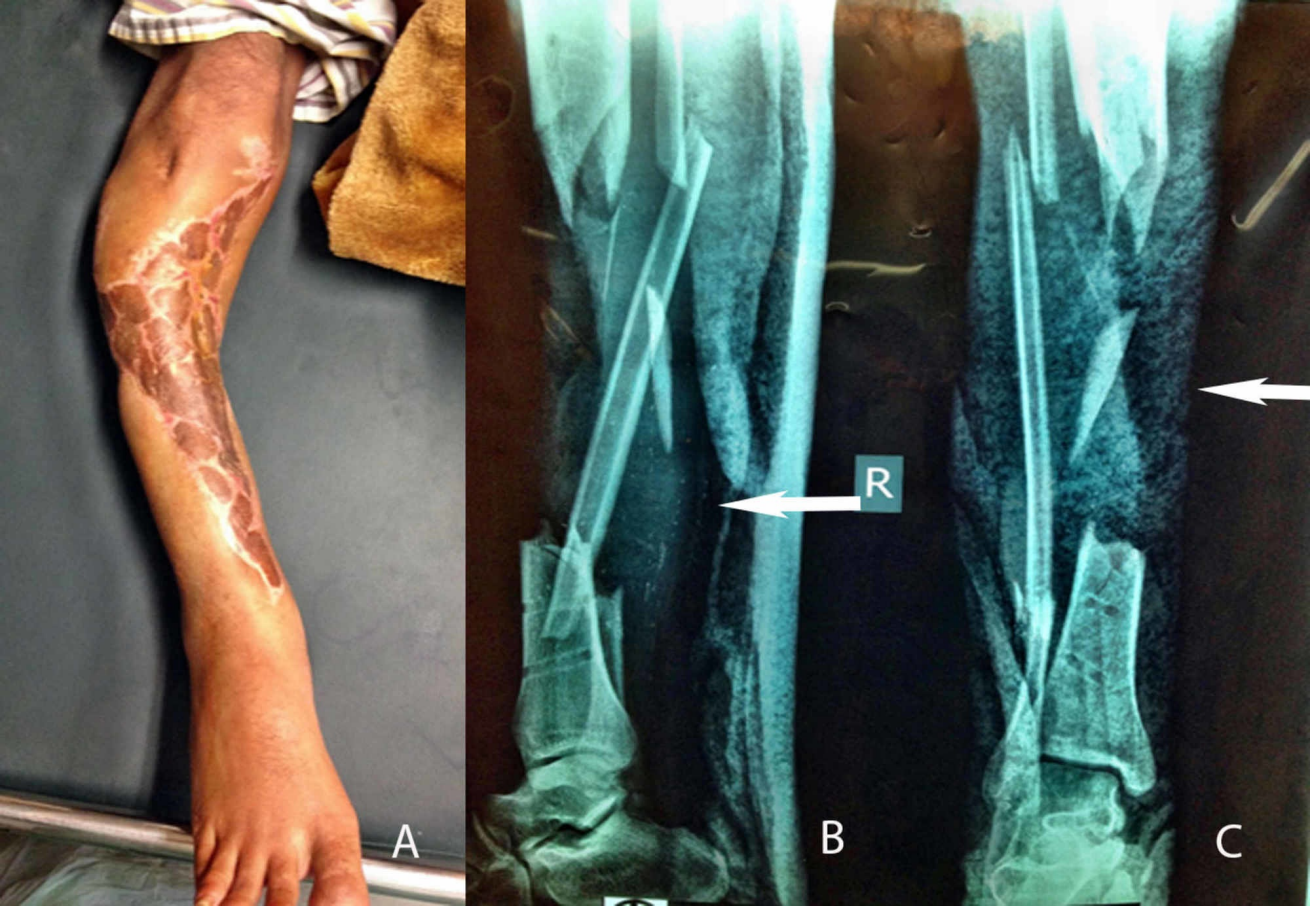
Preoperative pictures of post-traumatic bone defect with a live view (1A), lateral radiographic view (1B), and anteroposterior radiographic view (1C)

**Figure 2:**
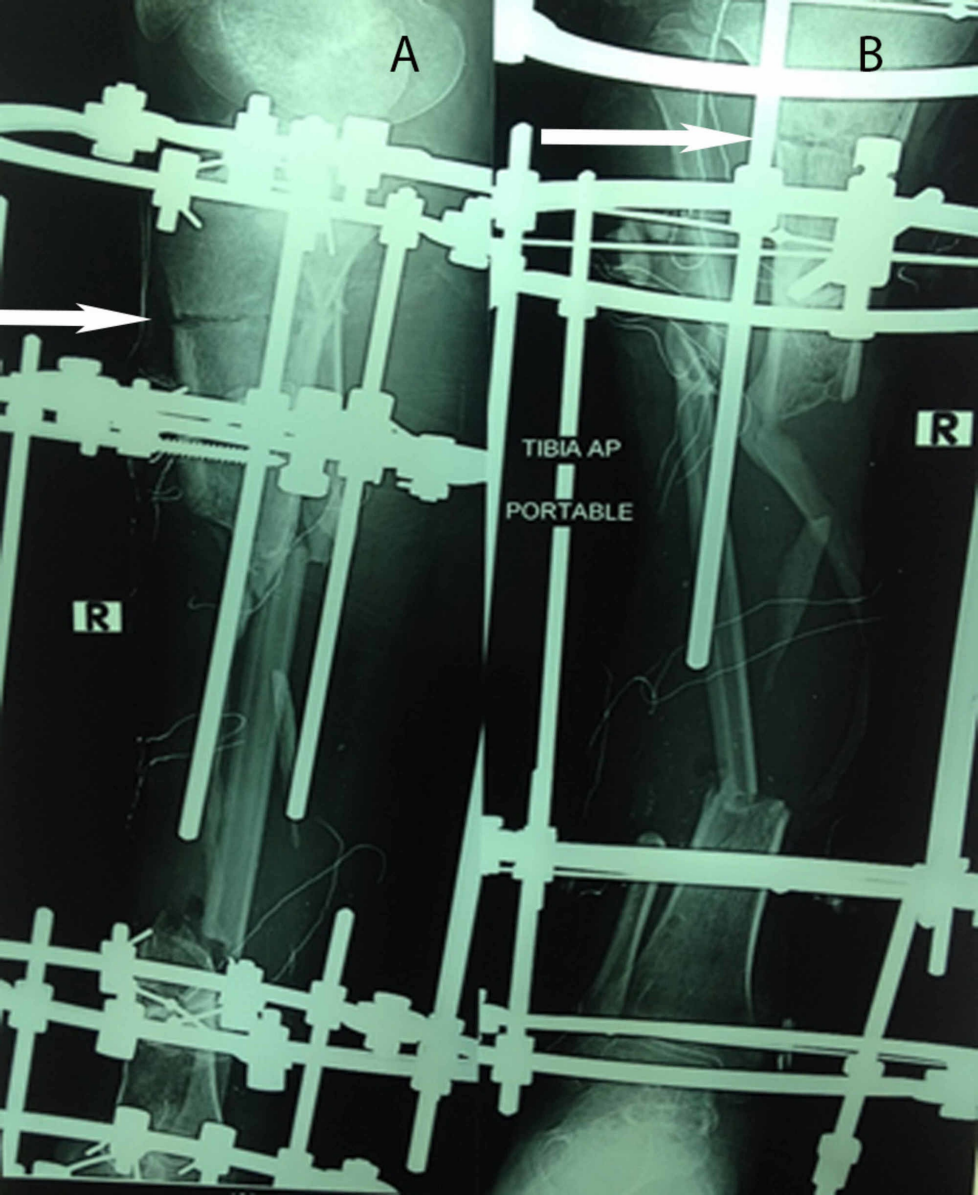
Immediate postoperative plain radiographs of the same patient who underwent multiple drill-hole osteotomy, with an anteroposterior (AP) view (2A) and a posteroanterior view (2B)

**Figure 3:**
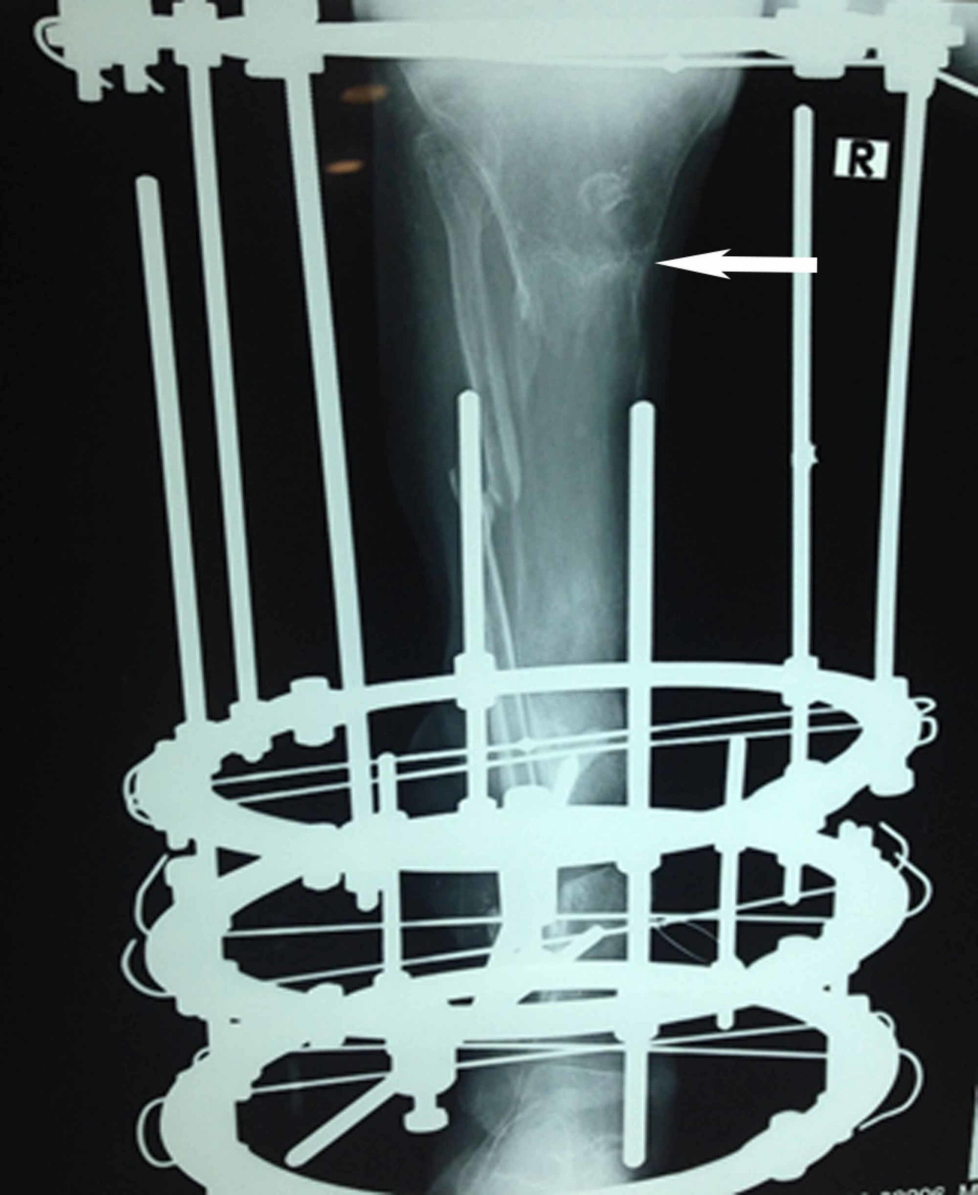
Postoperative plain radiograph of the same patient 13 months following the completion of bone transport showing the healed osteotomy site

**Figure 4:**
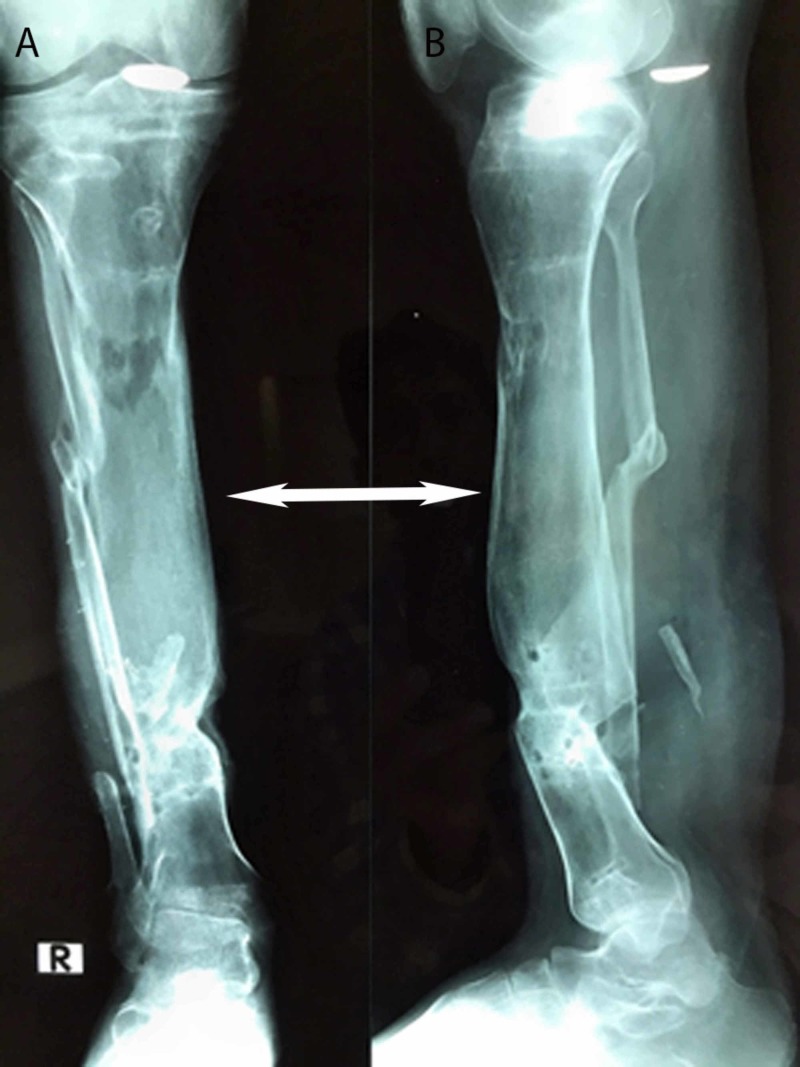
Postoperative plain radiographs of the same patient after removal of the Ilizarov frame, showing good regeneration of bone with an anteroposterior view (4A) and a lateral view (4B)

A total of 64 osteotomies were performed in 50 patients out of which 37 were males (74%) and 13 were females (26%) with a mean age of 33.14 ± 12 years. The patients were grouped into two: Group I was offered MDH osteotomy whereas Group II was offered a Gigli saw osteotomy. Overall, 38 osteotomies were performed at the proximal tibia and 26 were performed at the distal tibia (Table 1). Indications for which osteotomies were performed are mentioned in Table 1. A pictorial representation of the entire process in a single patient is given in Figures 1-4.

**Table 1:** Surgical characteristics of patients

Characteristics	n (%)
Number of tibial osteotomies (n=64)	
Gigli Saw	37 (58.8%)
Multiple drill-hole	27 (42.2%)
Location	
Proximal tibia	38 (59.4%)
Distal tibia	26 (40.6%)
Surgical indication	
Limb lengthening due to limb lengthening discrepancy	
Congenital	01 (2%)
Post-poliomyelitis	05 (10%)
Post-traumatic	11 (22%)
Bone transport due to bone defect	
Post-traumatic	21 (42 %)
Chronic osteomyelitis	09 (18%)
After bone tumor excision	03 (6%)

The overall mHI of both groups came out to be 1.60 ± 0.34 month/cm (range 1.0-2.5 month/cm). The mHI was compared between genders, the level of osteotomy, and both the techniques as shown in Table 2. When we compared the mHI of MDH and Gigli saw osteotomies, the mean mHI was 1.72 ± 0.33 month/cm (range 1.2-2.5 month/cm) and 1.54 ± 0.36 month/cm (range 1.0-2.5 month/cm) respectively. The healing index was significantly lower in the Gigli saw group.

**Table 2:** Comparison of modified healing indices of different parameters

Factor	Modified Healing Index	p-value
Overall both groups	1.60 ± 0.34 month/cm	
Technique of osteotomy		0.04
Multiple drill holes	1.72 ± 0.33 month/cm	
Gigli Saw	1.54 ± 0.36 month/cm	
Level of osteotomy		0.39
Proximal tibia	1.59 ± 0.34 month/cm	
Distal tibia	1.67 ± 0.38 month/cm	
Gender		0.42
Male	1.51 ± 0.39 month/cm	
Female	1.62 ± 0.43 month/cm	

None of our patients showed nonunion at osteotomy site. However, the problems of incomplete osteotomy in two cases and bone fractures in four cases were seen in multiple drill holes osteotomy. 

## Discussion

In the treatment of bone lengthening and bone transport, different types of low energy osteotomies have been described in the literature [8-12]. Gigli saw and MDH osteotomy has been recommended by many orthopedic surgeons because of better bone healing with minimal trauma to the soft tissue envelope at the osteotomy site [5-6,13]. Although many studies have been performed to evaluate their outcome, there is limited evidence to compare the two. Some advocate that there is high osseous regeneration in Gigli saw osteotomy [2,4,6], others report that there is no significant difference in bone healing between the two techniques [7].

Paktiss AS et al. performed Afghan percutaneous osteotomy in 50 tibias and >20 femurs and reported rapid healing, even with a relatively large amount of bone displacement. He recommended that Gigli saw osteotomy is safe, rapidly performed, and physiologically sound alternative to corticotomy [14]. In a similar study, Wardak MM et al. performed thousands of osteotomies using Gigli saw technique without facing any serious problems. According to him, the Gigli saw technique is minimally invasive, respects the periosteum, and is a low energy osteotomy that leaves a very smooth cut, and is especially important for rotational correction offering adequate regenerative properties [2].

Eralp L et al. [4] did a comparative study on two different osteotomy techniques for tibial lengthening and found that Gigli saw osteotomy patients had a less periosteal damage and significant better healing index compared with patients who underwent lengthening by MDH osteotomy (1.37 vs 1.9 months/cm), which again shows the biologic superiority of the Gigli saw osteotomy technique. However, in another comparative study of two proximal tibial osteotomy techniques by Peek AC et al., with 15 Gigli saw and 12 De Bastiani osteotomy techniques, there was no significant difference in healing indices of the two methods (2.2 vs 1.8 months/cm). Both Gigli saw and De Bastiani osteotomy techniques resulted in good bone formation following distraction osteogenesis [7].

In our study, the overall mHI of both groups was 1.60 ± 0.34 month/cm (range 1.0-2.5 month/cm). When the two techniques were compared, the mean mHI was 1.72 ± 0.33 month/cm (range 1.2-2.5 month/cm) in MDH group and 1.54 ± 0.36 month/cm (range 1.0-2.5 month/cm) in the Gigli saw group. The significantly low healing index of Gigli saw group (p = 0.04) is comparable to that published in many other studies and supports Gigli saw osteotomy over MDH osteotomy technique.

Limitations of this study include its retrospective analysis and small sample size. Also, the inclusion of only tibial bone osteotomies could lead to a selection bias.

The surgeons performing these procedures recommend that if Gigli saw is passed under the bone subperiosteally by two small incisions, this will minimize the local trauma and neurovascular injury and eliminate the possibility of incomplete osteotomy and unfavorable bone cracks. This can be another favourable point to employ the Gigli saw technique.

## Conclusions

After comparing the mHI of percutaneous Gigli saw osteotomy with that of MDH osteotomy performed in patients for bone lengthening or bone transport using Ilizarov fixator, we conclude that the former respects the periosteum and soft tissue envelope around the osteotomy more than the latter, resulting in better consolidation following distraction osteogenesis. Moreover, subperiosteal application of Gigli saw proved to be safe and effective.
